# Curaxin CBL0100 Blocks HIV-1 Replication and Reactivation through Inhibition of Viral Transcriptional Elongation

**DOI:** 10.3389/fmicb.2017.02007

**Published:** 2017-10-17

**Authors:** Maxime J. Jean, Tsuyoshi Hayashi, Huachao Huang, Justin Brennan, Sydney Simpson, Andrei Purmal, Katerina Gurova, Michael C. Keefer, James J. Kobie, Netty G. Santoso, Jian Zhu

**Affiliations:** ^1^Department of Microbiology and Immunology, University of Rochester Medical Center, Rochester, NY, United States; ^2^Incuron LLC, Buffalo, NY, United States; ^3^Department of Cell Stress Biology, Roswell Park Cancer Institute, Buffalo, NY, United States; ^4^Department of Medicine, Infectious Diseases, University of Rochester Medical Center, Rochester, NY, United States; ^5^Department of Biochemistry and Biophysics, University of Rochester Medical Center, Rochester, NY, United States

**Keywords:** human immunodeficiency virus (HIV), HIV latency, latency-promoting agents (LPA), FACT complex, curaxins

## Abstract

Despite combination antiretroviral therapy (cART), acquired immunodeficiency syndrome (AIDS), predominantly caused by the human immunodeficiency virus type 1 (HIV-1), remains incurable. The barrier to a cure lies in the virus' ability to establish a latent infection in HIV/AIDS patients. Unsurprisingly, efforts for a sterilizing cure have focused on the “shock and kill” strategy using latency-reversing agents (LRAs) to complement cART in order to eliminate these latent reservoirs. However, this method faces numerous challenges. Recently, the “block and lock” strategy has been proposed. It aims to reinforce a deep state of latency and prevent sporadic reactivation (“blip”) of HIV-1 using latency-promoting agents (LPAs) for a functional cure. Our studies of curaxin 100 (CBL0100), a small-molecule targeting the facilitates chromatin transcription (FACT) complex, show that it blocks both HIV-1 replication and reactivation in *in vitro* and *ex vivo* models of HIV-1. Mechanistic investigation elucidated that CBL0100 preferentially targets HIV-1 transcriptional elongation and decreases the occupancy of RNA Polymerase II (Pol II) and FACT at the HIV-1 promoter region. In conclusion, CBL0100 is a newly identified inhibitor of HIV-1 transcription that can be used as an LPA in the “block and lock” cure strategy.

## Introduction

The increase availability of combination antiretroviral therapy (cART) has significantly reduced HIV-1 associated morbidity and mortality (Deeks and Phillips, 2009; Ruelas and Greene, 2013). Nevertheless, cART is still unable to completely eliminate HIV-1 because cART-treated patients cannot clear the latently infected cells, mainly in resting memory CD4^+^ T cells. These viral reservoirs are long-lived, self-replenishing, and refractory to cART despite lowering plasma viral load to undetectable levels (Chomont et al., 2009; Darcis et al., 2017). Additionally, these viral reservoirs contribute to residual viremia during cART and viral rebound following cART interruption (Yukl et al., 2010). Latently infected CD4^+^ T cells are characterized as having transcriptionally silent but inducible replication-competent proviruses that can lead to the production of infectious virions when stimulated (Chun et al., 1997; Finzi et al., 1997; Wong et al., 1997). Therefore, there has been enormous focus on the “shock and kill” strategy, which aims to reactivate HIV-1 in latently infected CD4^+^ T cells and promote their elimination via viral cytopathic effects and/or immunological clearance, using small-molecule compounds called latency-reversing agents (LRAs). However, several roadblocks prevent this strategy from being effective (Darcis et al., 2017).

First, no LRA alone is unable to induce the sufficient viral cytopathic effects to eliminate HIV-1 cell reservoirs. Furthermore, cART-treated patients cannot mount an effective and robust HIV-specific cytotoxic T lymphocyte (CTL) response (Shan et al., 2012). Therefore, developing immunological strategies to enhance and sensitize patients' CTLs responses to reactivated cells are now believed to be needed for this approach to work (Archin et al., 2014). Moreover, It is also proposed that pro-apoptotic chemicals could be added to the “shock and kill” approach to sensitize the killing of HIV-infected cells via viral cytopathic effects (Badley et al., 2013; Cummins et al., 2016). However, this concept of “prime, shock, and kill” is still at the infant stage and targeting the apoptotic pathways could be toxic to healthy cells as well (Badley et al., 2013). Additionally, even the maximal stimulation of HIV latently infected cells using the most potent LRAs is unable to reactivate all replication-competent proviruses (Ho et al., 2013). All the tested LRAs or LRA combinations so far fail to surpass the global T cell stimulating agents, such as PMA/ionomycin and anti-CD3/CD8 antibodies (C^+^) to reactivate latent HIV (Bullen et al., 2014; Laird et al., 2015). In addition, Walker-Sperling et al. (2016) showed that certain LRA combinations, such as romidepsin (HDACi) and bryostatin-1 (PKC agonist), impairs the capability of HIV-specific CD8+ T cells to recognize and kill HIV-reactivated cells, which undoubtedly further hinder the success of “shock and kill” approach. Lastly, reactivating latently infected cells despite being in the context of cART context may lead to re-infection of tropic cells especially in sanctuary sites such as the brain where cART penetration is suboptimal (Ellis et al., 2007).

Recently, a strategy for a functional cure of HIV-1 has been proposed. Aptly named, the “block and lock” strategy aims to block the reactivation of latently infected HIV-1 proviruses, by using small-molecule inhibitors called latency-promoting agents (LPAs), so that a deep and irreversible latency is reached (Darcis et al., 2017). Interestingly, a few recent studies suggest that this strategy is quite feasible. Mousseau et al. characterized a Tat inhibitor, didehydro-coticostatin, which when combined with cART, leads to prolonged suppression of both HIV-1 transcription and viral production in latently infected cells (Mousseau et al., 2012, 2015). Similarly, Kim et al. reported that the anti-tumor peptide, G1V001, suppresses basal level HIV-1 transcription and viral reactivation through its inhibition of the NF-κB pathway in a Hsp90-dependent manner (Kim et al., 2016). Additionally, mTOR inhibitors have been implicated in the suppression of HIV-1 reactivation (Besnard et al., 2016). However, given that HIV-1 proviruses are highly heterogeneous in terms of sequences and integration sites, it will be necessary to identify new types of LPAs.

Our recent studies have identified the facilitates chromatin transcription (FACT) complex as a regulator of HIV-1 latency (Huang et al., 2015). FACT is composed of suppressor of Ty 16 (SUPT16H) and structure-specific recognition protein 1 (SSRP1), which acts as a histone chaperone that regulates the nucleosome assembly/disassembly complex and promotes RNA polymerase II (Pol II) mediated transcription (Belotserkovskaya and Reinberg, 2004). Interestingly, it was reported that FACT-inhibiting compounds, curaxins, suppress NF-κB-driven transcription in a FACT-dependent manner to elicit anti-tumor activity (Gasparian et al., 2011). Based on these observations, we postulated that curaxins might inhibit HIV-1 transcription and prevent HIV-1 reactivation as these processes are dependent on NF-κB activity (Ruelas and Greene, 2013). As supportive evidence, O'Connor et al. showed that targeting the FACT complex with curaxins inhibits the transcription and reactivation of human cytomegalovirus (HCMV)—another virus requiring NF-κB for proper transcription (O'Connor et al., 2016). Hence, we investigated a prototypical curaxin, curaxin 100 (CBL0100), as a potential HIV LPA.

## Materials and methods

### Compounds

CBL0100 was kindly provided by Cleveland Labs. Recombinant TNFα was (Cat. #BDB554618) purchased from Fisher Scientific. Prostratin (Cat. #P0077) was purchased from Sigma-Aldrich. Dimethyl sulfoxide (DMSO) (BP231-100) was purchased from Fischer Scientific.

### Antibodies

Mouse anti-RNA Pol II (Cat. #sc47701), anti-SUPT16H (Cat. #sc165987), and IgG (Cat. #sc2025) were obtained from Santa Cruz biotechnology. Anti-CD3 (Cat. #16003785) and anti-CD28 (Cat. #16029805) antibodies were purchased from Ebioscience.

### Viruses and plasmids

HIV-1 IIIB and HIV-1 NL4-3 strains were required from the NIH AIDS reagent program. VSV-G pseudotyped HIV-1 NL4-3 luciferase virus (HIV-1 Luc) was created by co-transfecting HEK293T cells with pCG-VSV-G and pNL4-3-Luc (ΔEnv). Transfection was performed using TransIT®-293 transfection reagent (Cat. #MIR 2700, Mirus), according to manufacturer's instruction. Envelope deleted DHIV-nef wild type (wt) and DHIV-nef NF-κB mutant (ΔκB) plasmids were kindly provided by Vicente Planelles (Bosque and Planelles, 2009). VSV-G-pseudotyped DHIV viruses were prepared as described above. Retroviruses expressing HIV-1 Tat (pQCXIP-Tat) were created by co-transfecting HEK293T cells with pQCXIP-Tat, pCG-VSV-G, and pCG-Gag/Pol as described previously (Huang et al., 2015; Power et al., 2015). pQCXIP empty vector was used as a negative control.

### Cell lines and culture

Jurkat T cell, JLTRG, J-LAT A2, J-LAT-A1, U1/HIV-1 cells were required from the NIH AIDS reagent program and cultured in RPMI-1640, 10% Fetal Bovine Serine (FBS), 1x penicillin-streptomycin media. Primary CD4^+^ T cells (Sanguine Biosciences, Lonza) and PBMCs were cultured in RPMI-1640, 20 mmol/L HEPES, 1x non-essential amino acids, and 1 × sodium butyrate. Primary CD4^+^ T cells and PBMCs were also supplied with IL2 (30 IU/ml) every 2 days. HEK293 were maintained in Dulbecco's Modified Eagle Medium with 10% FBS.

### Isolation of PBMCs from patient samples

HIV-1 positive, cART-treated, aviremic patients were recruited through the AIDS clinic at the Strong Memorial Hospital of University of Rochester Medical Center (Rochester, New York) to donate whole blood via leukapheresis. Study subjects were treated with ART for >3 years, had a suppressed viral HIV-RNA level (<50 copies/ml) for at least 6 months, and normal CD4^+^ T cell count (>300 cells/mm^3^). This study was approved by the University of Rochester Research Subjects Review Board (#RSRB00053667). PBMCs from three de-identified, HIV-1 positive, cART-naïve, viremic patients were provided by James Kobie at URMC. PBMCs from the whole blood of patients were isolated via the Ficoll-Hypaque gradient method. In brief, whole blood was diluted in 1x PBS (without Ca^2+^ or Mg^2+^) at a 1:1 ratio. Then 26 ml of the cell suspension was overlaid onto 14 ml of Ficoll-Paque (GE Healthcare) in a 50 ml conical tube. Samples were then centrifuged for 20 min at 966 × g (without braking and at room temperature). Next, the buffy coat band from the interface was collected and transferred to a new sterile 50 ml conical tube. PBMCs were then washed three times using 1x PBS with 5 mM EDTA. Cells rested in the complete RPMI media in the presence of Nevirapine (600 nM) for 3 days and CD8^+^ T cells were depleted by negative selection using the CD8 MicroBeads (Miltenyi Biotec) following the manufacturer's instruction.

### Cell viability assay

Viability was assessed by measuring ATP levels following CBL0100 treatment using Promega Cell-Titer-Glo Luminescence cell viability kit (G7570), following the manufacturer's instructions. The luciferase signal was measured using the Cytation 5 imaging reader (BioTek).

### p24 ELISA assay

*S*upernatants from HIV-1 infected primary CD4^+^ T cells or Jurkat T cells were collected. The protocol of p24 assay provided by the manufacturer (ABL Science) was used to determine viral p24 production (pg/ml) with a minor modification in wash procedure: five washes of 280 μl 1x wash buffer whenever indicated.

### Flow cytometry

J-LAT clones were seeded at 4 × 10^6^ cells/ml. Cells were treated with DMSO or stimulated with 10 ng/ml TNFα, or 1 μM Prostratin. Cells were also co-treated with DMSO or CBL0100 (0.1 μM). Cells were kept in culture for 24 h. Viable cells were then sorted using an Accuri C6 flow cytometer (BD Biosciences). Percentage of GFP+ cells were analyzed by using Flow Jo. The same procedure was used for THP89GFP cells.

### IC_50_ of CBL0100 to inhibit HIV-1 replication

Fifty nanograms of p24 of NL4-3 (X4) was used to infect 1 × 10^6^ cells in the presence of DMSO or CBL0100 at a series of concentrations. Cells were washed once with 1 × PBS, re-suspendended in fresh media, and re-treated with CBL0100. At 48 h post-infection, supernatant was collected and the viral load was quantified by p24 ELISA assay.

### Primary CD4^+^ T cell model of HIV-1 latency

The Planelles model of HIV-1 latency was used to generate latently infected CD4^+^ T cells with slight modifications (Bosque and Planelles, 2009; Hayashi et al., 2017; Huang et al., 2017). Briefly, naïve CD4^+^ T cells were isolated from two healthy PBMC donors and were stimulated with anti-CD3/CD28 antibodies that were precoated on a Nunc-Immuno Maxi Sorp plate (Thermo Scientific). Cells were incubated with complete medium supplemented with 10 ng/ml of TGFβ, 2 μg/ml of anti-humanIL-12, and 1 μg/ml of anti-human IL-4 (R&D Systems) for 3 days. At day 7, cells were infected with HIV-1 Luc and allowed to revert back to a quiescent/latent state. At day 11, cells were stimulated with anti-CD3/CD28 (+) or mock stimulated with DMSO (−) and co-treated with CBL0100 (0.1 μM) or DMSO for another 3 days. Luciferase signal was measured using One Glo luciferase assay kit (Promega) following the manufacturer's instructions.

### Chromatin immunoprecipitation-qPCR

ChIP assays followed this previously described protocol (Laspia et al., 1989) with minor changes. Briefly, 1 × 10^6^ U1/HIV-1 cells were activated with 10 ng/ml TNFα, or mock activated with DMSO, and co-treated with either DMSO or CBL0100 (0.1 uM). At 24 h, the cells were cross-linked using 0.5% formaldehyde. After quenching the cross-linking reaction with glycine (125 mM), the cells were washed with cold 1 × PBS and lysed in 1 × CE buffer (10 mM HEPES-KOH, pH 7.9, 60 mM KCl, 1 mM EDTA, 0.5% Nonidet P-40, 1 mM DTT, and protease inhibitor mixture). Cell lysate was centrifuged at 700 × g for 10 min at 4°C to pellet the nuclei. The nuclei pellet was resuspended in 1 × SDS lysis buffer (1% SDS, 10 mM EDTA, 50 nM Tris-HCl, pH 8.1, and protease inhibitor mixture) to a final concentration of 3 × 10^7^ cells/ml, and the nuclear lysate was sonicated for 2 min using a Fisher Scientific™ model 505 sonic dismembrator (1-s on and 1-s off cycles at 50% impulse) to fragment DNA to an average size of ~500 bp. Cellular debris was spun down, and the supernatant was diluted 10-fold with 1 × ChIP dilution buffer (0.01% SDS, 1% Triton X-100, 1.2 mM EDTA, 16.7 mM Tris-HCl, pH 8.1, 150 nM NaCl, and protease inhibitor mixture) and incubated with 5 μg of antibodies against SUPT16H, RNA Pol II (CTD4H8) or control antiserum for overnight with rotation at 4°C. Fifty microliters of protein A/G beads were pre-equilibrated with 0.5 mg/ml BSA (Fisher Scientific) and 0.125 mg/ml calf thymus DNA (Trevigen) for 2 h at 4°C and then added to each sample, which were incubated for another 2 h. Beads were collected and then washed once with each of the following: low salt buffer (0.1% SDS, 1% Triton X-100, 2 mM EDTA, 20 mM Tris-HCl, pH 8.1, 150 mM NaCl), high salt buffer (0.1% SDS, 1% Triton X-100, 2 mM EDTA, 20 mM Tris-HCl, pH 8.1, 500 mM NaCl), and LiCl buffer (0.25 M LiCl, 1% Nonidet P-40, 1% sodium deoxycholace, 1 mM EDTA, 10 mM Tris-HCl, pH 8.1). Beads were then washed twice with 1 × TE buffer (10 mM Tris-HCl, pH 8.1, 0.1 mM EDTA). Immunoprecipitated protein-DNA complexes were eluted twice with fresh elution buffer (1% SDS, 0.1 M NaHCO_3_) for 1.25 h at room temperature. Eluates and nuclear lysates (“input”) were heated at 65°C for overnight, to reverse cross-links, in the presence of 0.2 M NaCl. Samples were then treated with 1 μl of 20 mg/ml proteinase K (Life Technologies), 10 μl of 2 M Tris-HCl (pH 6.5), and 10 μl of 0.5 M EDTA for 2 h at 50°C. Released DNA was extracted with phenol/chloroform, precipitated by ethanol, and then resuspended in 100 μl of water. One to three microliters of each sample was used for qPCR using primer sets for amplifying the nuc-1 region of LTR. The qPCR was performed on the CFX Connect™ real time PCR detection system (Bio-Rad), in a 20-μl volume using the following program: 95°C for 1 min and 50 cycles of 95°C for 15 s and 60°C for 30 s.

### qPCR assay to measure HIV-1 transcription

Quantitative PCR was used to measure the levels of different HIV-1 transcripts as described previously (Zhu et al., 2012). Briefly, 5 × 10^5^ U1/HIV-1 cells were activated with 10 ng/ml TNFα, or mock activated with DMSO, and co-treated with either DMSO or CBL0100 (0.1 μM). Cells were then collected and subjected to mRNA extraction (RNeasy® Mini Kit, Qiagen) and reverse transcription (iScript™ cDNA Synthesis Kit, Bio-Rad). qPCR was conducted using iTaq™ Universal SYBR® Green Supermix (Bio-Rad) on a CFX Connect™ Real-Time PCR System (Bio-Rad), in a 20 μl volume using the following program: 95°C for 1 min and 40 cycles of 95°C for 15 s and 60°C for 30 s. The following qPCR primers were used: initiation, elongation-1, elongation-2, and *gag* mRNA (see Table S1). GAPDH was used as a reference gene. The same qPCR method was used to quantify HIV-1 *gag* mRNA levels in DHIV-nef viruses infected Jurkat cells.

### Nested qPCR assay to quantify HIV-1 viral load

Nested qPCR assay to quantify HIV-1 viral load was done as described previously (Mousseau et al., 2015). Specifically, the supernatant of cells subjected to HIV-1 infection or HIV-1 reactivation was collected, and the viral RNA was extracted using a QIAmp® Viral RNA kit (Qiagen) following the manufacturer's instruction. The RNA samples were treated with DNase I (Invitrogen) for 10 min at 25°C, and then inactivated by EDTA at 65°C for 10 min. Reverse transcription coupled qPCR assay was carried out using the Superscript® III One-Step RT-PCR System with Platinum® Taq High Fidelity (Life Technologies) in a total volume of 50 μl. The HIV-1 *gag*-specific primers P1 and P2 were used for PCR with the following thermal cycles: 50°C for 30 min, 94°C for 2 min, 16 cycles 94°C for 15 s, 62°C for 30 s, 68°C for 60 s, and a final elongation step at 65°C for 5 min. The pre-amplified RT-PCR products were purified via QIAquick® PCR Purification Kit (Qiagen) and a second nested qPCR assay was conducted using TaqMan® Universal PCR Master Mix (Applied Biosystems) in a 25 μl reaction. P3, P4 primers and probe were used for the second PCR. Cycling conditions included UNG incubation (50°C for 2 min), followed by initial denaturation (95°C, 10 min), 45 cycles of amplification (95°C, 15 s; 50°C, 20 s; 60°C, 1 min). All PCR assays were conducted on a CFX connect™ real time PCR detection system (Bio-Rad). A serial dilution of HIV-1 IIIB virus of known concentration was used to create a standard curve for the absolute quantification.

### Data analysis and statistics

Statistical analysis was performed using Student's *t*-test or one-way analysis of variance (ANOVA) followed by Tukey's multiple-comparison test (GraphPad Prism 5.0 software). A *P*-value of < 0.05 was considered statistically significant. Dose response curve was created by nonlinear regression model and the 50% inhibitory concentrations (IC_50_) was calculated using GraphPad Prism 5.0 software.

## Results

### CBL0100 inhibits acute HIV-1 replication

**Figure 1:**
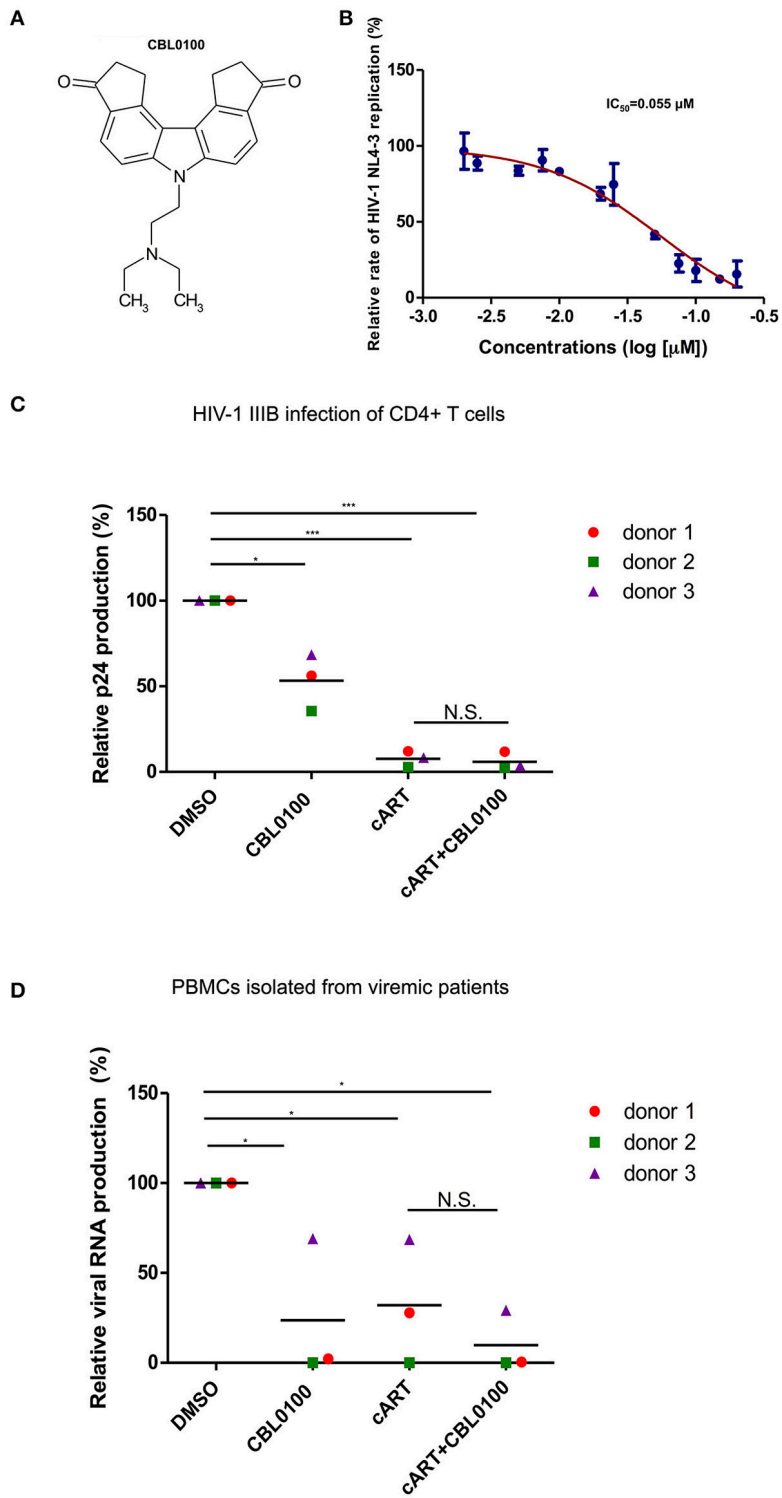
CBL0100 inhibits the acute replication of HIV-1. **(A)** Structure of Curaxin 100 (CBL0100). **(B)** Titration curve of CBL0100's effect on the acute replication of HIV-1 NL4-3 virus in Jurkat cells. Data is represented as the percentage of inhibition normalized to DMSO (0.1%). IC_50_ is calculated using Graph Pad Prism equation. Standard error bars represent data from two independent experiments. **(C)** Activated primary CD4^+^ T cells from three healthy donors were infected with HIV-1 IIIB in the presence of 0.1% DMSO, 0.1 μM CBL0100, cART (0.2 μM Raltegravir, 0.18 μM AZT, 0.1 μM Efavirenz), or cART+CBL0100. At 3 dpi, viral load was assessed by p24 ELISA and represented as percent of DMSO (grand mean, ^*^p < 0.05, ^**^p < 0.01, ^***^p < 0.001, student *t*-test). **(D)** CD8-depleted PBMCs from three HIV-positive, cART naïve, viremic patients were cultured in the presence of DMSO, 0.1 μM CBL0100, cART, or cART+CBL0100. At 3 dpi, viral load was assessed by nested *gag* qPCR and represented as percent of DMSO. Statistical analysis is similar as in panel **(C)**. NS, Not Significant.

Presumably, an ideal LPA should also block acute HIV-1 replication, which may still exist in HIV-positive, cART-treated, aviremic patients, and contributes to residual viremia and/or reservoir replenishment (Darcis et al., 2017). We thus first determined the inhibitory effect of CBL0100 on acute HIV-1 replication *in vitro* (Figure 1A). The p24 ELISA assay of cell supernatant showed that CBL0100 efficiently inhibits HIV-NL4-3 replication in Jurkat cells in a dose-dependent manner (IC_50_ = 0.055 μM) (Figure 1B). Cell viability was also measured for CBL0100-treated Jurkat cells, and showed that there was either no or mild cytotoxicity at tested concentrations (0.05, 0.1, 0.2 μM) (Figure S1A). CBL0100 was used at the concentration of 0.1 μM for all subsequent experiments unless otherwise indicated. We next determined the inhibitory effect of CBL0100 on HIV-1 IIIB replication in primary CD4^+^ T cells isolated from three healthy donors. At 3 days post-of-infection (dpi), CBL0100 alone moderately decreased the p24 level in the supernatant of HIV-infected CD4^+^ T cells across the three donors tested, without any cytotoxicity (Figure 1C, Figure S1B). However, the inhibitory effect of CBL0100 almost disappeared at 5 dpi (data not shown). cART potently inhibited viral replication at both time points and was not enhanced by the addition of CBL0100 (Figure 1C). Furthermore, we determined the inhibitory effect of CBL0100 using PBMCs isolated from three HIV-positive, cART-naïve, viremic donors. Overall, CBL0100 alone significantly reduced the viral RNA level in the supernatant of cultured PBMCs across all donors (Figure 1D). Specifically, it had a greater inhibitory effect on donors 1 and 2, while its effect on donor 3 was mild (Figure 1D). The addition of CBL0100 further enhanced cART to block HIV-1 replication for donors 1 and 3 but not donor 2, as cART alone was already potent for donor 2. Collectively, these data suggest that in certain individuals when used in combination with cART, CBL0100 can generate an intensified effect and quickly control viral output during acute infection.

### CBL0100 inhibits HIV-1 reactivation in latency cell lines

**Figure 2:**
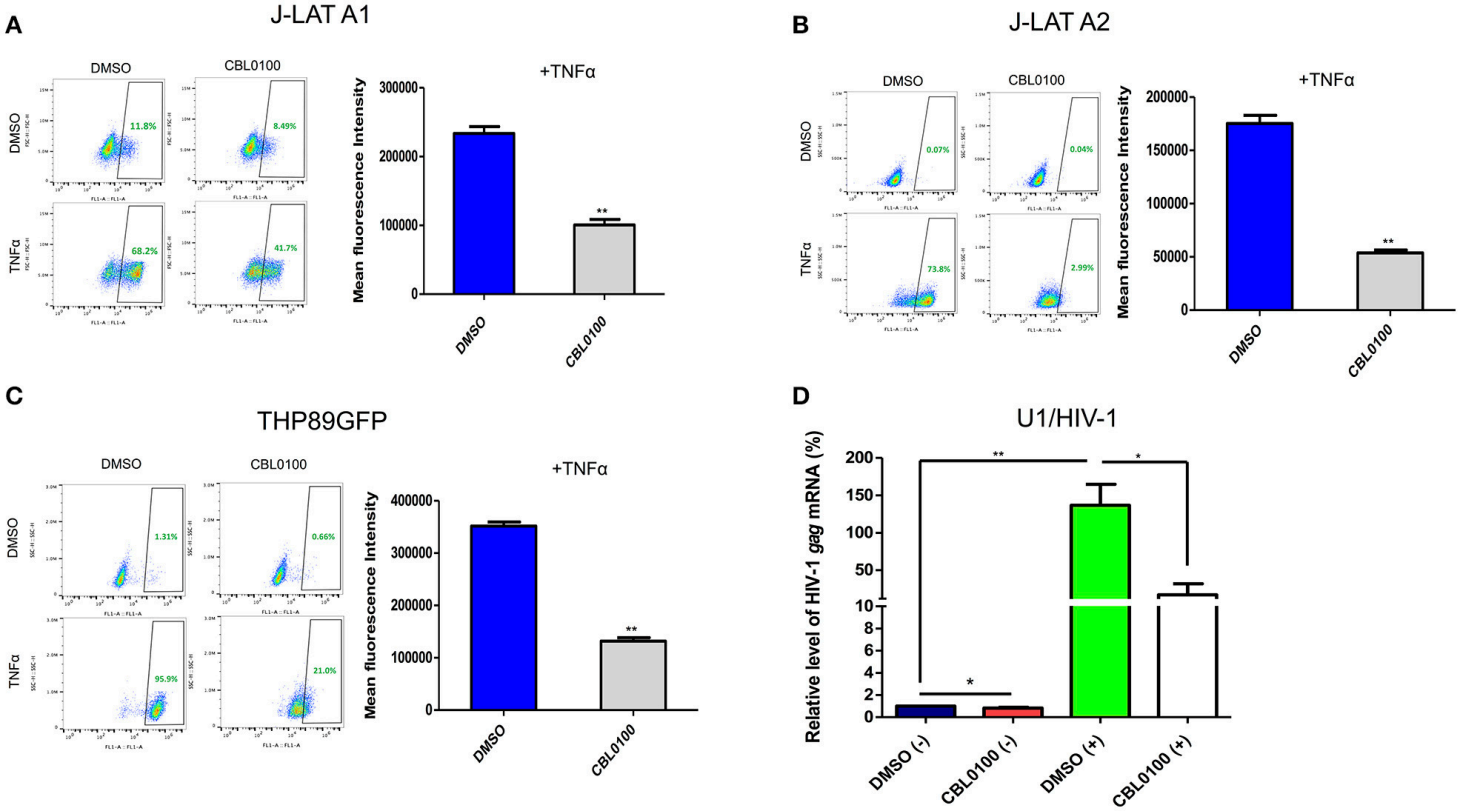
CBL0100 blocks HIV-1 reactivation in latency cell lines. **(A)** J-LAT-A1 cells were treated with TNFα (10 ng/ml) or mock treated in the presence of 0.1 μM CBL0100 or 0.1% DMSO for 24 h. GFP^+^ cells were sorted by flow cytometry. Mean fluorescence intensity (MFI) is measured in GFP^+^ cells and results are shown on right. Data obtained from three independent experiments (mean ± s.e.m., ^*^p < 0.05, ^**^p < 0.01, ^***^p < 0.001, Student *t*-test). **(B)** J-LAT-A2 cells were subjected to the same assay as in **(A)**. **(C)** THP89GFP cells were subjected to the same assay as in **(A)** except that 0.2 μM CBL0100 was used. **(D)** U1/HIV-1 cells were treated with 10 ng/ml TNFα (+) or mock treated (–) and co-treated with either 0.1% DMSO or 0.1 μM CBL0100 for 24 h. Total mRNA was extracted and subjected to the qPCR assay that measures the mRNA level of HIV-1 *gag*. GAPDH was used as the reference. The normalized mRNA level of HIV-1 *gag* from all tested samples are represented as relative to DMSO (–). Data is the average of three independent experiments (mean ± s.e.m., ^*^p < 0.05, ^**^p < 0.01, ^***^p < 0.001, student *t*-test).

We next decided to determine whether CBL0100 inhibits the reactivation of latently infected HIV-1 proviruses, given that viral reservoirs are a major concern for HIV/AIDS patients (Archin et al., 2014). Two J-LAT cell lines, A1 and A2, were stimulated with TNFα to reactivate latent HIV-1, which was determined by measuring GFP expression. CBL0100 treatment (0.1 μM) led to a decrease in both the GFP+ cell population and GFP mean fluorescence intensity (MFI) in both J-LAT A1 and A2 cells without any cytotoxicity; however, its effect on A2 cells was more striking (Figures 2A,B, Figure S2C). No significant effect of CBL0100 was observed in un-stimulated cells.

In addition to memory CD4^+^ T cells, cells from the monocyte/macrophage lineage may be another source of HIV-1 latent reservoirs (Kumar et al., 2014). We further determined the inhibitory effect of CBL0100 in two monocytic cell line models of HIV-1 latency, THP89GFP and U1/HIV-1 cells. For THP89GFP cells, CBL0100 treatment at the higher but non-toxic concentration (0.2 μM) potently blocked TNFα-induced HIV-1 reactivation, which was observed by measuring GFP+ cell population and MFI (Figure 2C, Figure S2D). For U1/HIV-1 cells, CBL0100 treatment (0.1 μM) resulted in a remarkable decrease of intracellular HIV-1 gag mRNA following TNFα stimulation without any cytotoxicity (Figure 2D, Figure S2E). Furthermore, we determined whether CBL0100 blocks HIV-1 reactivation through the use of different stimulators. We found that CBL0100 inhibited prostratin-induced HIV-1 reactivation in both J-LAT A2 and THP89GFP cells. Additionally, the suppressive effect of CBL0100 on prostratin-induced cells was similar to that of TNFα-induced cells (Figures 2B,C, Figure S2A,B). Together, these results suggest that CBL0100 is a potent LPA, which inhibits HIV-1 reactivation triggered by multiple stimulators in multiple types of reservoir cells.

### CBL0100 inhibits HIV-1 reactivation in primary model systems

**Figure 3:**
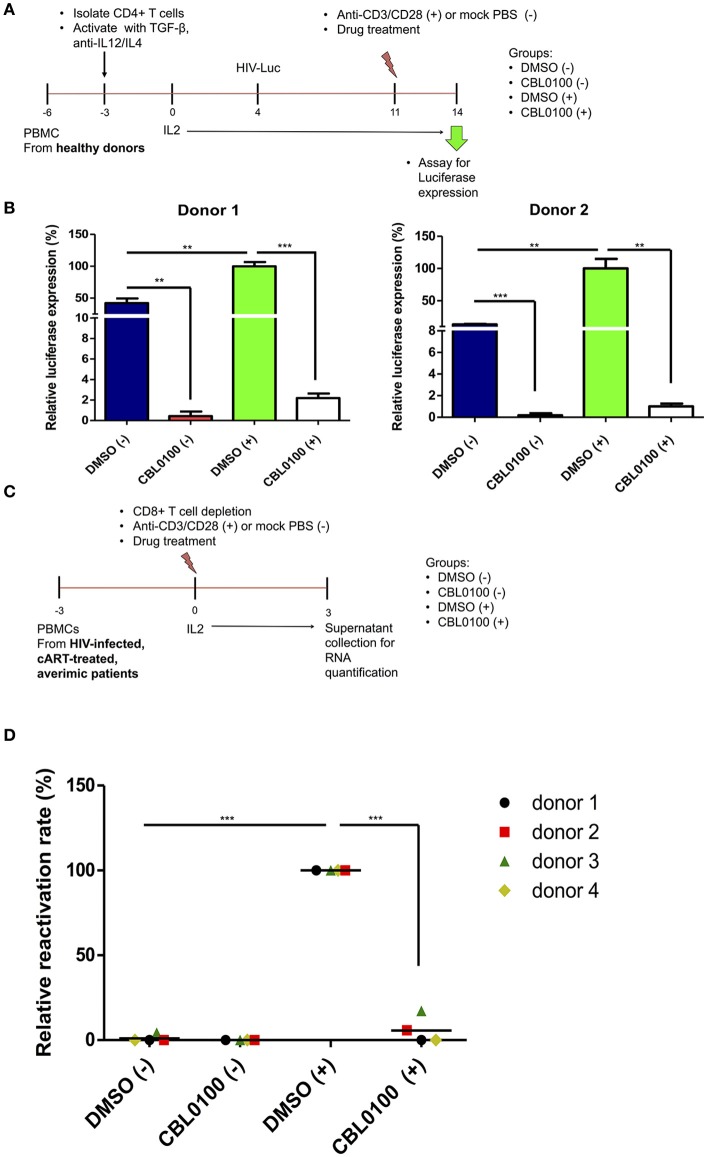
CBL0100 blocks HIV-1 reactivation from latency in primary cells. **(A)** The scheme to test CBL0100's effect on HIV-1 reactivation by using a primary CD4^+^ T cell model (Planelles' model) of HIV-1 latency. **(B)** The HIV-1 latently infected primary CD4^+^ T cells were treated with anti-CD3/CD28 antibodies (+) or mock treated with PBS (–) in the presence of 0.1 μM CBL0100 or 0.1% DMSO for 3 days. The intracellular luciferase signals from all tested samples were measured and represented as relative to DMSO (+) for two healthy donors (mean ± s.d., ^*^p < 0.05, ^**^p < 0.01, ^***^p < 0.001, student *t*-test). **(C)** The scheme to test CBL0100's effect on HIV-1 reactivation by using the CD8-depeleted PBMCs that were isolated from four HIV-positive, cART-treated, aviremic patients. **(D)** CD8-depeleted PBMCs were treated with anti-CD3/CD28 antibodies (+) or mock treated with PBS (–) in the presence of 0.1 μM CBL0100 or 0.1% DMSO for 3 days. The viral RNA level in the supernatant from all tested samples was determined by qPCR and represented as relative to DMSO (+) for the four different donors (grand mean, ^*^p < 0.05, ^**^p < 0.01, ^***^p < 0.001, student *t*-test).

To further verify the potency of CBL0100 with respect to inhibiting HIV-1 reactivation, we moved our studies from cell lines to primary cells. First, we tested CBL0100 by using a primary CD4^+^ T cell model of HIV-1 latency previously described by Planelles' group (Bosque and Planelles, 2009). In this model, HIV-1 latency is established in non-polarized, memory CD4^+^ T cells infected with VSV-G pseudo-typed HIV-luciferase virus (Figure 3A). HIV-1 reactivation was induced by treating these cells with anti-CD3/CD28 antibodies (+). CBL0100 treatment (0.1 μM) potently inhibited HIV-1 reactivation in anti-CD3/CD28 stimulated CD4^+^ T cells isolated from two healthy donors, which was determined by measuring the intracellular luciferase activity (Figure 3B). CBL0100 treatment also showed similar effects in un-stimulated cells, indicating that it can also block the basal transcription of HIV-1 proviruses. These results, using primary cells, support our hypothesis that CBL0100 can be used as an LPA to block the reactivation of HIV in latent reservoir cells.

To further validate these observations, we examined the effects of CBL0100 on HIV-1 reactivation in latent reservoir cells using PBMCs isolated from HIV-positive, cART-treated, aviremic patients. Specifically, we depleted CD8^+^ T cells from the PBMCs of four HIV-positive, cART-treated, aviremic donors and stimulated them with C+ for a period of 3 days (Figure 3C). CBL0100 (0.1 μM) treatment strikingly suppressed HIV-1 reactivation, reaching ~95% reduction of viral RNA output as compared to the DMSO control (+) for all donors, while presenting minimal cytotoxicity (Figure 3D, Figure S4). Individual suppression ranged from 83 to 99.9% for these donors (Figure S3). On the other hand, un-stimulated cells did not produce a measurable viral RNA output, which is likely due to the resting state of these cells.

### CBL0100 inhibits HIV-1 transcriptional elongation

**Figure 4:**
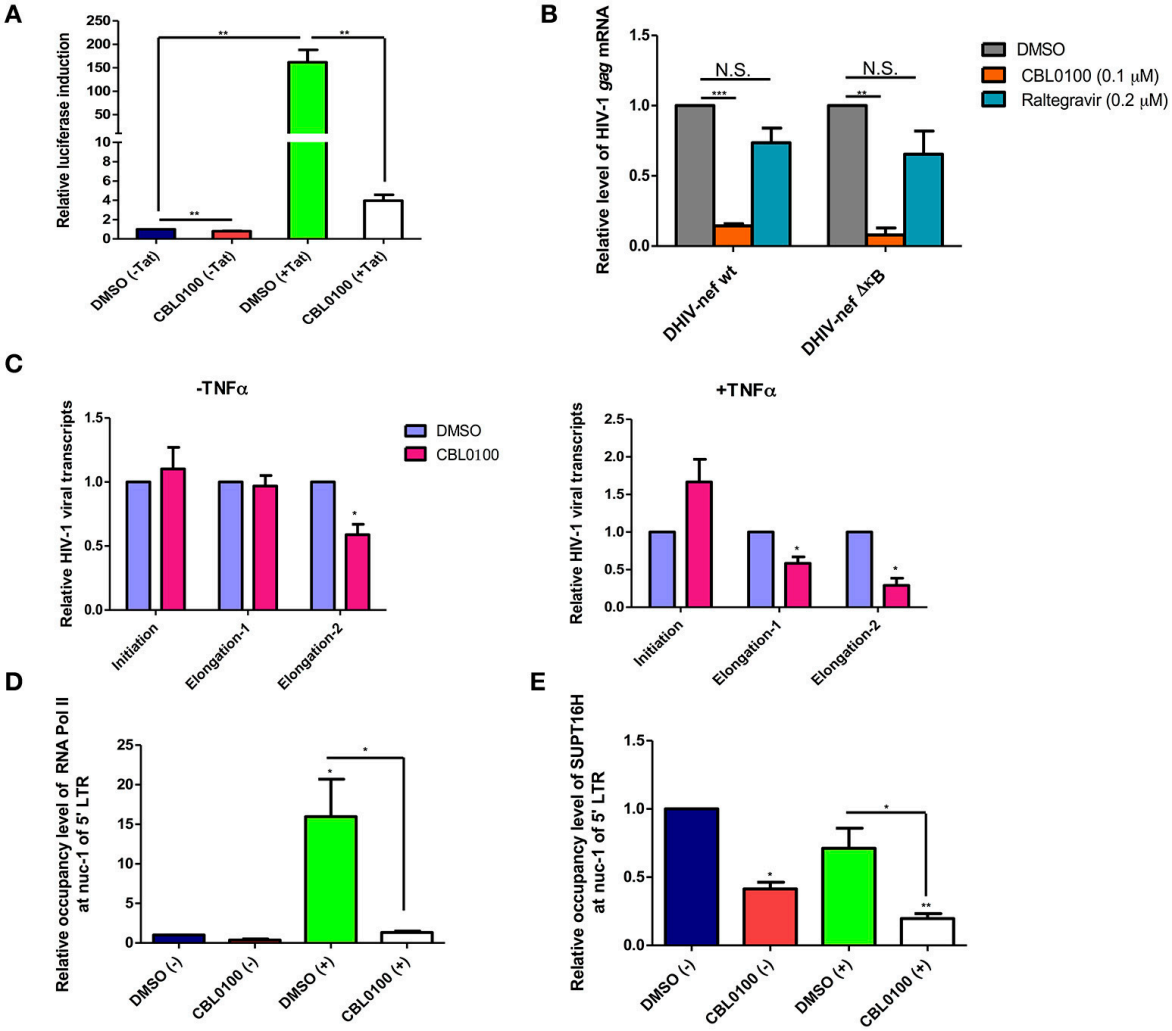
CBL0100 suppresses HIV-1 transcriptional elongation by reducing the LTR occupancy of RNA Pol II and FACT. **(A)** TZM-bl cells were pre-treated with 0.1% DMSO or 0.1 μM CBL0100 for 6 h, followed by transduction of either pQCXIP-empty (–Tat) or pQCXIP-Tat retroviral vectors. At 48 h, luciferase signal was measured. Data is normalized to DMSO (–Tat) and obtained from three independent experiments (mean ± s.e.m., ^*^p < 0.05, ^**^p < 0.01, ^***^p < 0.001, student *t*-test). **(B)** Jurkat cells were infected with VSV-G-DHIV-nef either wt or ΔκB viruses for 24 h. Cells were then treated with 0.1% DMSO, 0.1 μM CBL0100 or 0.2 μM raltegravir for additional 24 h. HIV-1 *gag* mRNA levels were measured by qPCR. GAPDH was used as the reference. The normalized mRNA level of HIV-1 gag transcripts from all tested samples are represented relative to the corresponding DMSO control and data is from two independent experiments (mean ± s.e.m., *p* < 0.05, student *t*-test). **(C)** U1/HIV-1 cells were treated with 10 ng/ml TNFα (+) or un-treated (–) in the presence of 0.1 uM CBL0100 or 0.1% DMSO for 24 h. Initiated and elongated HIV-1 transcripts were measured by qPCR. Two different sets of primers were used for qPCR to measure the elongated transcripts in reference to the different distance downstream of 5′ LTR promoter. GAPDH was used as the reference. The normalized mRNA level of HIV-1 transcripts from all tested samples were represented as relative to the DMSO control (mean ± s.e.m. *p* < 0.05, student's *t*-test). **(D,E)** U1/HIV-1 cells were treated similar as in **(A)** and subjected to the ChIP-PCR assays for RNA Pol II **(D)** and SUPT16H **(E)**. Antibodies against RNA Pol II or SUPT16H were used for the immunoprecipitations of protein/DNA complexes. The mIgG antibody was used as the negative control. The co-precipitated DNA species were extracted and subjected to the qPCR assay by using the primer set that amplifies the nuc-1 region of HIV-1 5′ LTR. The background signal (mIgG) was subtracted from tested samples and the normalized DNA level of nuc-1 region is represented as relative to DMSO (–). Results represent one of two independent experiments (mean ± s.d., ^*^p < 0.05, ^**^p < 0.01, ^***^p < 0.001, One-way ANOVA followed by tukey post-test).

Earlier studies of CBL0100 have indicated that it interferes with NF-κB activity through the inhibition of FACT complex (Gasparian et al., 2011). We investigated whether CBL0100 elicits its antiretroviral effect on HIV-1 replication and reactivation through a similar mechanism. We first examined the effects of CBL0100 on HIV-1 5′ LTR promoter activity with or without HIV-1 Tat transactivator, in TZM-bl cells which harbor an integrated LTR-luciferase construct. At the basal level (–Tat), there was a modest reduction of luciferase expression between DMSO and CBL0100 treated cells (Figure 4A). However, in the presence of Tat (+Tat) CBL0100 treatment led to a ~97% reduction of luciferase expression as compared to DMSO (Figure 4A). These results confirm that CB0L100 indeed inhibits the HIV-1 Tat-LTR mediated viral transcription. We next tested whether the inhibitory effect of CBL0100 on HIV-1 transcription is dependent on the NF-κB binding site of the LTR. We determined the drug effect of CBL0100 on the viral expression of VSV-G pseudo-typed HIV-1 DHIV-nef viruses that contain either a wild-type (wt) or a mutated NF-κB binding site (ΔκB) in Jurkat cells (Bosque and Planelles, 2009) (Figure 4B). Interestingly, CBL0100 showed a similar inhibitory effect on the wt and ΔκB viruses, suggesting that CB0100 does not require an intact NF-κB site at the 5′ LTR to suppress HIV-1 transcription; which is different from its anti-tumor activity (Gasparian et al., 2011). For this assay, we also included raltegravir as a negative control, as it does not inhibit post-integrated steps of HIV-1 life cycle.

To further understand how CBL0100 blocks HIV-1 reactivation through inhibition of viral transcription, we turned to the U1/HIV-1 latency model. We measured the different HIV-1 transcripts (initiated or elongated) using specific primer sets in TNFα-stimulated, CBL0100-treated U1/HIV-1 cells. Surprisingly, CBL0100 treatment (0.1 μM) slightly increased the initiated transcript in TNFα-stimulated cells but has no obvious effect in un-stimulated cells (Figure 4C). However, CBL0100 reduced the elongated transcripts in both TNFα-stimulated and un-stimulated cells, with more significant effects observed in TNFα stimulated cells—particularly when comparing the longer elongated transcripts (Figure 4C). These data suggest that CBL0100 may specifically target HIV-1 transcriptional elongation vs. initiation. HIV-1 transcriptional elongation is mainly mediated by RNA polymerase II (Pol II), which overcomes pausing at the nucleosome-1 (nuc-1) region of the HIV-1 5′ LTR promoter (Laspia et al., 1989; Kim et al., 2002). Therefore, we sought to determine the effect of CBL0100 on the occupancy of Pol II at nuc-1 in TNFα-stimulated U1/HIV-1 cells, through the use of a chromatin immunoprecipitation (ChIP) assay. Elongated HIV-1 transcripts are less efficiently produced at basal level and in the absence of Tat (Laspia et al., 1989), while TNFα stimulation enhances their production. Our results consistently showed that TNFα stimulation greatly increased the Pol II occupancy at nuc-1, but CBL0100 treatment (0.1 μM) dramatically blocked such increase (Figure 4D). At the basal condition, without TNFα stimulation, Pol II occupancy at nuc-1 also seemed to be reduced following CBL0100 treatment. Furthermore, Gaspian et al. has previously shown that both Pol II and FACT proteins are depleted at the promoter region of NF-κB dependent genes in TNFα-stimulated, CBL0100-treated tumor cells, and that their transcription is also severely down-regulated (Gasparian et al., 2011).

We next determined the effect of CBL0100 on the occupancy of SUPT16H, a FACT protein, at nuc-1 in TNFα-stimulated U1/HIV-1 cells using another ChIP assay. Consistently, we identified that the occupancy of SUPT16H at nuc-1 substantially decreased with CBL0100 treatment (0.1 μM) at both the basal level and following TNFα stimulation (Figure 4E). Overall, our mechanistic studies suggest that CBL0100 inhibits HIV-1 transcriptional elongation by reducing the occupancy of both Pol II and FACT complex at the 5′ LTR promoter region of HIV-1.

## Discussion

Several groups including ours recognized that the FACT complex is an important host factor that plays a role in regulating HIV-1 acute infection, as well as HIV-1 latency (Huang et al., 2015; Lopez et al., 2016). Curaxins, small molecule compounds, target the activity of the FACT complex, and elicit an anti-tumor activity (Gasparian et al., 2011). Given these earlier studies our primary goal was to investigate whether curaxins also possess antiretroviral activity through their targeting of the FACT complex. We first examined whether curaxins were able to block the acute infection of HIV-1. We found that the curaxin CBL0100 elicits a certain inhibitory effect on the acute infection of HIV-1, although such effect varies between two *in vitro* cell models. Using primary CD4^+^ T cells, infected with HIV-1 IIIB, we found that CBL0100 alone generally had a moderate retroviral effect across three HIV-naïve donors. However, the addition of CBL0100 to cART renders no further beneficial effect—probably due to the maximal efficacy of cART treatment to block acute infection of HIV-1 in this model (Figure 1C). Using PBMCs isolated from HIV-positive, cART-naïve, viremic patients, we observed that the use of CBL0100 alone was sufficient to potently reduce the viral load in two of our three donors (Figure 1D). The antiretroviral effect of CBL0100 alone was moderate in the third donor, but cART alone was also moderate and the addition of CBL0100 to cART resulted in a significant enhancement in this case (Figure 1D). Our conclusion from these results is that when used alone CBL0100 may heterogeneously block the acute infection of HIV-1 across different individuals. Nevertheless, the addition of CBL0100 to a cART regimen may enhance its antiretroviral effect, particularly under certain circumstances where the use of cART alone is inefficient to block the acute infection of HIV-1.

Acute infection of HIV-1 is normally well controlled by cART. However, HIV-1 latent reservoirs are persistent even in the presence of cART. Thus, the current challenge for a functional cure of HIV-1 is to eliminate the sporadic reactivation (“blip”) from latently infected HIV-1 proviruses, and the potential reseeding of HIV-1 latent reservoirs. Since there are no drug components in cART that block reactivated HIV-1 transcription, it is necessary to develop new LPAs to block HIV-1 transcription, in order to induce a deep and irreversible HIV-1 latency for the “block and lock” cure strategy. Our results convincingly show that CBL0100 alone potently blocks HIV-1 reactivation in multiple HIV-1 latency cell models with better efficacy than its inhibitory effect on the acute infection of HIV-1. In fact, CBL0100 alone was able to block HIV-1 reactivation in all tested latency cell lines (J-LAT-A1/A2, THP189GFP, and U1/HIV-1) induced using different LRAs (TNFα, prostratin), without obvious cytotoxicity (Figure 2, Figure S2). Interestingly, CBL0100 alone significantly decreased the MFI in HIV-reactivated, GFP-positive J-LAT A1 cells but only moderately reduced the percentage of GFP-positive cells (Figure 2A), this is probably due to leaky HIV-1 reactivation in these cells where the environment more mimics acute replication. More profound and convincing validations of curaxins as potential LPAs were performed using primary cell models, as the HIV-1 latency cell lines discussed previously, were constructed primarily using cancer cells, which do not physiologically recapitulate the nature of HIV-1 latent reservoir cells (Archin et al., 2014). HIV-1 reactivation was induced using anti-CD3/CD28 antibodies, which is presumed to be one of the strongest stimulators (Darcis et al., 2015). In the Planelles model of HIV-1 latency that is established in primary memory CD4^+^ T cells (Bosque and Planelles, 2009; Chomont et al., 2009), CBL0100 alone almost completely blocked both basal and induced viral transcription (Figures 3A,B). This finding could be significant as it indicates that a certain level of low-level transcription existing in HIV-1 latently infected cells could be blocked by CBL0100 alone; alternatively, CBL0100 may also block the weak HIV-1 reactivation from reservoir cells that can be sporadically triggered by certain signals, such as TNFα (Pace et al., 2011). Similar results were observed using CD8-depleted PBMCs isolated from four HIV-positive, cART-treated, aviremic patients, which presumably contain natural HIV-1 reservoir cells. This *ex vivo* testing suggests that it could be promising to use CBL0100 alone as a potential LPA to reinforce HIV-1 latency in a clinical setting.

**Figure 5:**
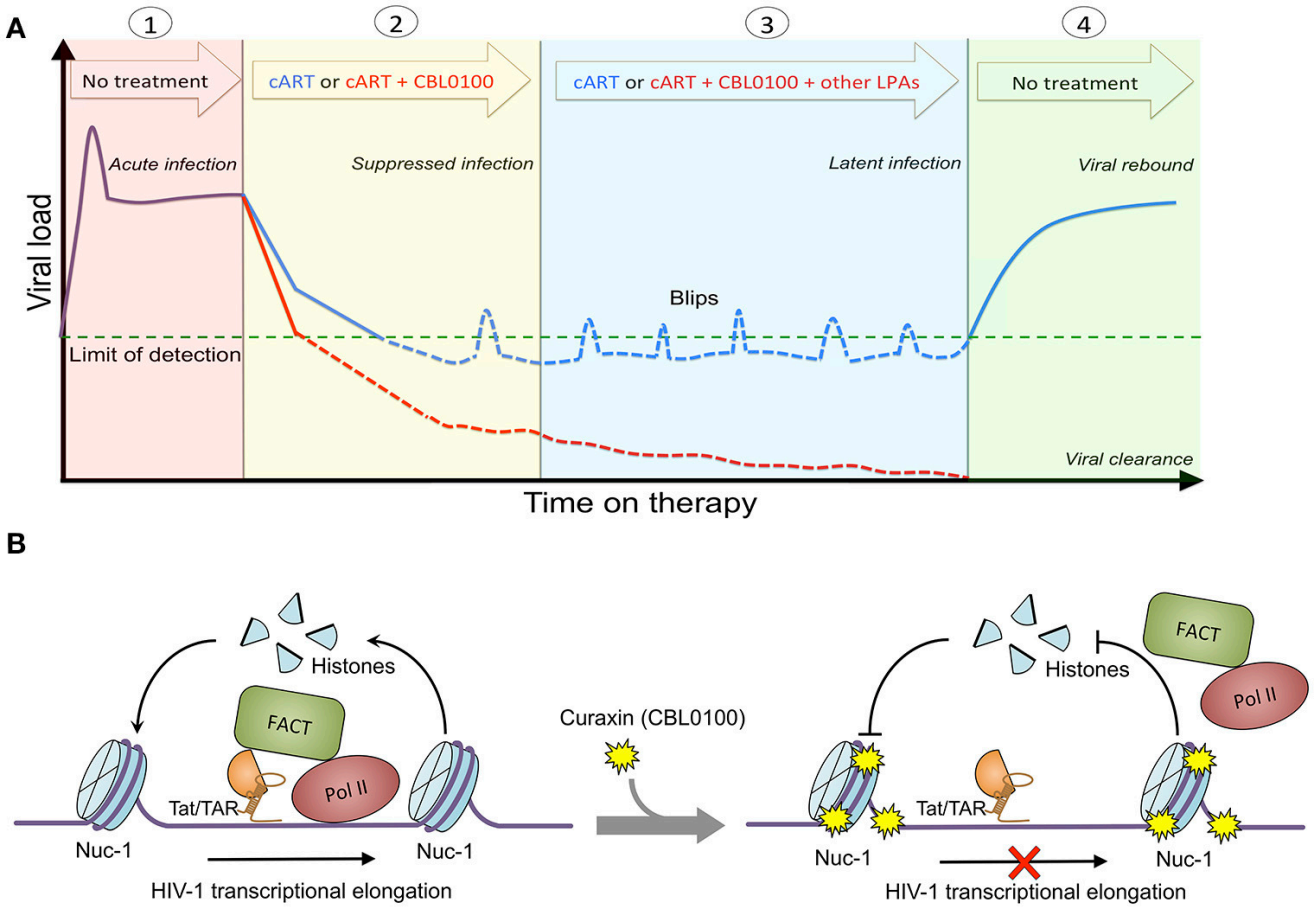
Proposed treatment regimen and molecular mechanism of CBL0100. **(A)** Proposed treatment regimen for the use of CBL0100 in combination with cART for the “block and lock” cure strategy. (1) Acute infection: primary infection of HIV-1 peaks but decreases to stable level when the immune system recognizes the virus (no treatment). (2) Suppressed infection: addition of CBL0100 to cART may intensify cART and lead to faster control of viremia and smaller size of latent reservoirs at the baseline (red line: cART + CBL0100; blue line: cART). (3) Latent infection: addition of CBL0100 and/or other LPAs to cART may prevent the spontaneous viral reactivation (blips) and continue the reduction of reservoir size (red line: cART + CBL0100 and/or other LPAs; blue line: cART). (4) Viral clearance (vs. rebound): addition of CBL0100 and/or other LPAs to cART may eventually reinforce a deep and irreversible silencing of HIV-1 proviruses and lead to the depletion of viral reservoirs for a functional cure (no treatment). **(B)** Proposed molecular mechanism of CBL100 in suppression of HIV-1 transcription. HIV-1 Tat associates with FACT and recruits it in the proximity to nucleosome at nuc-1. FACT facilites the disassembly and reassembly of the nucleosome at nuc-1 to allow the RNA Pol II to pass for transcriptional elongation. However, in the presence of CBL0100 it intercalates into chromatins and blocks the FACT accessibility/association with nucleosome at nuc-1. RNA Pol II is unable to pass and dissociate from nuc-1. HIV-1 transcription elongation is thus terminated by CBL0100.

CBL0100 acts as a promising antiretroviral compound, to block both HIV-1 replication and reactivation, which prompted us to explore its molecular mechanism of action and possibly gain insights that could be useful to further improve its' drug effects. We first demonstrated that CBL0100 potently suppresses Tat-LTR mediated HIV-1 transcription in the TZM-bl cells (Figure 4A). We further found that surprisingly the NF-κB binding site is dispensable with respect for CB0100's ability to inhibit HIV transcription, which is different from its anti-tumor activity (Gasparian et al., 2011) (Figure 4B). In a U1/HIV-1 latency cell model, we were able to further pinpoint that CBL0100 preferentially affects the elongated vs. initiated transcripts, particularly in TNFα-treated cells (Figure 4C). These result are consistent and make sense: first, at the basal level without TNFα induction elongated transcripts are not efficiently produced, thus CBL0100 has less of an impact; second, CBL0100 does not require the ΔκB site that is required for the initiation of HIV-1 transcription but not the elongation step (Figure 4B) (Ruelas and Greene, 2013). Additionally, CBL0100 also reduces the occupancy of RNA Pol II at the nuc-1 region of the 5′ HIV-LTR that represents a pivotal site for HIV-1 transcription to switch from initiation to elongation (Nabel and Baltimore, 1987; Laspia et al., 1989; Garber et al., 1998) (Figure 4D). Interestingly, earlier studies indicated that CBL0100 intercalates into chromatin DNA and leads to a decrease of RNA Pol II mediated transcription, in tumor cells through a parallel decrease of FACT recruitment (Gasparian et al., 2011). In our studies, CBL0100 indeed significantly reduced the occupancy of SUPT16H at the nuc-1 region of the 5′ LTR in both TNFα-treated and un-treated cells (Figure 4E). It seems that induction by TNFα does not increase the occupancy of SUPT16H, but significantly increases the occupancy of RNA Pol II at nuc-1. Thus, we postulate that in the case of TNFα-induced HIV-1 reactivation, CBL0100 could directly inhibit the association of FACT at nuc-1 by intercalating into the viral chromatin DNA, effectively preventing the nucleosome disassembly as well as the association of RNA Pol II at the nucleosome and its function in transcriptional elongation (Figure 5B), although TNFα treatment did allow for more recruitment of RNA Pol II at the nuc-1 region, this is probably through a FACT-independent mechanism (Ott et al., 2011). In contrast, at the basal level the occupancy of nuc-1 by RNA Pol II is low, which may explain why CBL0100 in general has less of an inhibitory effect on HIV-1 transcription in un-treated vs. TNFα-induced cells, despite CBL0100's capability to deplete the occupancy of SUPT16H at nuc-1 (Figures 3B, 4C–E). In fact, this could be the common mechanism for curaxins to regulate the expression of targeted genes, as Gaspian et al also showed similar effects of curaxins on the recruitment of FACT proteins and RNA Pol II at the promoters of NF-κB regulated genes when they examined the antitumor activity of curaxins (Gasparian et al., 2011). The antiretroviral and antitumor effects of CBL0100 have a similar end effect on Pol II and FACT chromatin association, but they are slightly different in terms of the NF-κB dependence, this requires further characterization (Figure 4B). In addition, we noticed that inhibition of SUPT16H using CBL0100 generates a different consequence as compared to its knockdown using RNAi. Specifically, our earlier studies showed that RNAi-mediated depletion of SUPT16H moderately enhanced HIV-1 transcription and reactivation (Huang et al., 2015). On the other hand, our current studies show that in general, CBL0100 blocks overall HIV-1 transcription, although CBL0100 slightly increases HIV-1 initiated transcripts (Figure 4C). The likely explanation for this phenomenon is that inhibition of SUPT16H using CBL0100 and depletion of SUPT16H using RNAi may generate different patterns of SUPT16H redistribution across the HIV-1 5′ LTR region so that the outcome of transcription could be different, given that the FACT complex is a nucleosome remodeler that can both disassembly and reassemble nucleosomes when RNA Pol II passes (Belotserkovskaya and Reinberg, 2004).

Overall, we identified curaxin CBL0100 as a new class of LPA. We postulate that treatment of this compound would prevent the proper recruitment of FACT and RNA Pol II at the HIV 5′ LTR, and thereby block RNA Pol II mediated transcriptional elongation of HIV-1, which is independent of the NF-κB pathway (Figure 5B). Although CBL0100 alone moderately blocks the acute infection of HIV-1, CBL0100 may intensify the antiretroviral potency of cART. Furthermore, CBL0100 alone is already sufficient to potently block HIV-1 reactivation induced by several strong LRAs. Given to these two attributes, we propose the hypothetical treatment regimen using CBL0100 shown in Figure 5A: CBL0100 could be used in combination with cART for a faster control of viral load in viremic patients; CBL0100 alone or in addition with other LPAs could be used to block HIV-1 reactivation from latency and reduce the size of viral reservoirs in aviremic patients under the control of cART. Ultimately, we hope that HIV-1 latent reservoirs would be significantly reduced and permanently sealed to a point where patients would no longer require any additional cART therapy (Figure 5A). However, the detailed plan for this combinatory treatment will need to be further investigated, especially in *in vivo* animal models, such as HIV-1 humanized mice models and/or SIV macaque models (Denton et al., 2011; Archin et al., 2014). Lastly, it will be interesting to further evaluate whether there would be a synergistic effect between CBL0100 and other reported LPAs, such as GV001 peptide (Kim et al., 2016) or mTOR inhibitors (Besnard et al., 2016). This may offer a maximal effect to deeply silence the sequence-diversified HIV-1 proviruses (Darcis et al., 2017).

## Author contributions

JZ conceived the project. JZ and MJ analyzed the results, designed the study, and wrote the paper. MJ, TH, and JB conducted the experiments. HH, SS, NS, AP, and KG provided reagents and advised the study. MK and JK recruited the HIV/AIDS averimic and viremic patients for this study, respectively.

### Conflict of interest statement

The authors declare that the research was conducted in the absence of any commercial or financial relationships that could be construed as a potential conflict of interest.

## Supplementary material

Figure S1 **(A)** The cytotoxicity test of CBL0100 at three selected concentrations (0.05, 0.1, and 0.2 μM). Data was obtained from two independent experiments and compared to DMSO (mean ± s.e.m., ^*^*p* < 0.05, ^**^*p* < 0.01, ^***^*p* < 0.001, student *t*-test). **(B)** Cell viability of healthy primary CD4^+^ T cells treated with CBL0100 at 0.1 μM for 3 days. Data is normalized to DMSO control (mean ± s.d., ^*^*p* < 0.05, ^**^*p* < 0.01, ^***^*p* < 0.001, student *t*-test).

Figure S2 **(A)** J-LAT-A2 cells were stimulated with prostratin (1 μM) or mock treated with DMSO in the presence of CBL0100 (0.1 μM) or DMSO for 24 h. The percentage of GFP+ cells was measured by flow cytometry. MFI of GFP+ cells was also measured. Data are from three independent experiments (mean ± s.e.m., ^*^*p* < 0.05, ^**^*p* < 0.01, ^***^*p* < 0.001, Student *t*-test). **(B)** The same experiment as in panel A was performed for THP89GFP cells. 0. 5 μM Prostratin and 0.2 μM CBL0100 were used. **(C)** Cell viability of J-LAT A2 following 24-h treatment with 0.1 μM CBL0100. Data is normalized to DMSO control and from three independent experiments (mean ± s.e.m., ^*^*p* < 0.05, ^**^*p* < 0.01, ^***^*p* < 0.001, student *t*-test). **(D)** The same experiment as in panel C was performed for THP89GFP cells. 0.2 μM CBL0100 were used. **(E)** The same experiment as in **(C)** was performed for U1/HIV-1 cells.

Figure S3 CD8-depeleted PBMCs were stimulated with anti-CD3/CD28 (+) or mock treated with PBS (–) in the presence of 0.1 μM CBL0100 or DMSO for 3 days. The viral RNA level in the supernatant from all tested samples was determined by qPCR and represented as relative to DMSO (+) for respective donors. Effect of CBL0100 on individual donors was measured from triplicate measurements (mean ± s.d., ^*^*p* < 0.05, ^**^*p* < 0.01, ^***^*p* < 0.001, student *t*-test). ND, Not detected.

Figure S4 Cell viability of CD8-depeleted PBMCs from four HIV-positive, cART-treated donors following the CBL0100 treatment with the anti-CD3/CD28 antibodies induction (+) or the mock treatment (–). ATP levels was assessed for each conditions and data was normalized to the corresponding DMSO control.

Table S1 Primer sequences for the qPCR assays.

## References

[B1] Archin N. M. Sung J. M. Garrido C. Soriano-Sarabia N. Margolis D. M. (2014). Eradicating HIV-1 infection: seeking to clear a persistent pathogen. Nat. Rev. Microbiol. 12, 750–764. 10.1038/nrmicro3352 25402363 PMC4383747

[B2] Badley A. D. Sainski A. Wightman F. Lewin S. (2013). Altering cell death pathways as an approach to cure HIV infection. Cell Death Dis. 4:e718. 10.1038/cddis.2013.248 23846220 PMC3730421

[B3] Belotserkovskaya R. Reinberg D. (2004). Facts about FACT and transcript elongation through chromatin. Curr. Opin. Genet. Dev. 14, 139–146. 10.1016/j.gde.2004.02.004 15196460

[B4] Besnard E. Hakre S. Kampmann M. Lim H. W. Hosmane N. N. Martin A.. (2016). The mTOR complex controls HIV latency. Cell Host Microbe 20, 785–797. 10.1016/j.chom.2016.11.001 27978436 PMC5354304

[B5] Bosque A. Planelles V. (2009). Induction of HIV-1 latency and reactivation in primary memory CD4^+^ T cells. Blood 113, 58–65. 10.1182/blood-2008-07-168393 18849485 PMC2614643

[B6] Bullen C. K. Laird G. M. Durand C. M. Siliciano J. D. Siliciano R. F. (2014). New *ex vivo* approaches distinguish effective and ineffective single agents for reversing HIV-1 latency *in vivo*. Nat. Med. 20, 425–429. 10.1038/nm.3489 24658076 PMC3981911

[B7] Chomont N. El-Far M. Ancuta P. Trautmann L. Procopio F. A. Yassine-Diab B.. (2009). HIV reservoir size and persistence are driven by T cell survival and homeostatic proliferation. Nat. Med. 15, 893–900. 10.1038/nm.1972 19543283 PMC2859814

[B8] Chun T.-W. Carruth L. Finzi D. Shen X. DiGiuseppe J. A. Taylor H.. (1997). Quantification of latent tissue reservoirs and total body viral load in HIV-1 infection. Nature 387, 183–188. 10.1038/387183a0 9144289

[B9] Cummins N. W. Sainski A. M. Dai H. Natesampillai S. Pang Y.-P. Bren G. D.. (2016). Prime, shock, and kill: priming CD4 T cells from HIV patients with a BCL-2 antagonist before HIV reactivation reduces HIV reservoir size. J. Virol. 90, 4032–4048. 10.1128/JVI.03179-15 26842479 PMC4810548

[B10] Darcis G. Kula A. Bouchat S. Fujinaga K. Corazza F. Ait-Ammar A.. (2015). An in-depth comparison of latency-reversing agent combinations in various *in vitro* and *ex vivo* HIV-1 latency models identified bryostatin-1+ JQ1 and ingenol-B+ JQ1 to potently reactivate viral gene expression. PLoS Pathog. 11:e1005063. 10.1371/journal.ppat.1005063 26225566 PMC4520688

[B11] Darcis G. Van Driessche B. Van Lint C. (2017). HIV latency: should we shock or lock? Trends Immunol. 38, 217–228. 10.1016/j.it.2016.12.003 28073694

[B12] Deeks S. G. Phillips A. N. (2009). Clinical review: HIV infection, antiretroviral treatment, ageing, and non-AIDS related morbidity. BMJ 338, 288–292. 10.1136/bmj.a3172 19171560

[B13] Denton P. W. Olesen R. Choudhary S. K. Archin N. M. Wahl A. Swanson M. D.. (2011). Generation of HIV latency in BLT humanized mice. J. Virol. 86, 630–634. 10.1128/JVI.06120-11 22013053 PMC3255928

[B14] Ellis R. Langford D. Masliah E. (2007). HIV and antiretroviral therapy in the brain: neuronal injury and repair. Nat. Rev. Neurosci. 8, 33–44. 10.1038/nrn2040 17180161

[B15] Finzi D. Hermankova M. Pierson T. Carruth L. M. Buck C. Chaisson R. E.. (1997). Identification of a reservoir for HIV-1 in patients on highly active antiretroviral therapy. Science 278, 1295–1300. 10.1126/science.278.5341.1295 9360927

[B16] Garber M. Wei P. Jones K. (1998). HIV-1 Tat interacts with cyclin T1 to direct the P-TEFb CTD kinase complex to TAR RNA. Cold Spring Harb. Lab. Press 63, 371–380. 10384302 10.1101/sqb.1998.63.371

[B17] Gasparian A. V. Burkhart C. A. Purmal A. A. Brodsky L. Pal M. Saranadasa M.. (2011). Curaxins: anticancer compounds that simultaneously suppress NF-κB and activate p53 by targeting FACT. Sci. Transl. Med. 3:95ra74. 10.1126/scitranslmed.3002530 21832239 PMC6281439

[B18] Hayashi T. Jean M. Huang H. Simpson S. Santoso N. G. Zhu J. (2017). Screening of an FDA-approved compound library identifies levosimendan as a novel anti-HIV-1 agent that inhibits viral transcription. Antiviral Res. 146, 76–85. 10.1016/j.antiviral.2017.08.013 28842263 PMC5654649

[B19] Ho Y.-C. Shan L. Hosmane N. N. Wang J. Laskey S. B. Rosenbloom D. I.. (2013). Replication-competent noninduced proviruses in the latent reservoir increase barrier to HIV-1 cure. Cell 155, 540–551. 10.1016/j.cell.2013.09.020 24243014 PMC3896327

[B20] Huang H. Liu S. Jean M. Simpson S. Huang H. Merkley M.. (2017). A novel bromodomain inhibitor reverses HIV-1 latency through specific binding with BRD4 to promote tat and P-TEFb association. Front. Microbiol. 8:1035. 10.3389/fmicb.2017.01035 28638377 PMC5461361

[B21] Huang H. Santoso N. Power D. Simpson S. Dieringer M. Miao H.. (2015). Fact proteins, SUPT16H and SSRP1, are transcriptional suppressors of HIV-1 and HTLV-1 that facilitate viral latency. J. Biol. Chem. 290, 27297–27310. 10.1074/jbc.M115.652339 26378236 PMC4646377

[B22] Kim H. Choi M.-S. Inn K.-S. Kim B.-J. (2016). Inhibition of HIV-1 reactivation by a telomerase-derived peptide in a HSP90-dependent manner. Sci. Rep. 6:28896. 10.1038/srep28896 27363520 PMC4929463

[B23] Kim Y. K. Bourgeois C. F. Isel C. Churcher M. J. Karn J. (2002). Phosphorylation of the RNA polymerase II carboxyl-terminal domain by CDK9 is directly responsible for human immunodeficiency virus type 1 Tat-activated transcriptional elongation. Mol. Cell. Biol. 22, 4622–4637. 10.1128/MCB.22.13.4622-4637.2002 12052871 PMC133925

[B24] Kumar A. Abbas W. Herbein G. (2014). HIV-1 latency in monocytes/macrophages. Viruses 6, 1837–1860. 10.3390/v6041837 24759213 PMC4014723

[B25] Laird G. M. Bullen C. K. Rosenbloom D. I. Martin A. R. Hill A. L. Durand C. M.. (2015). *Ex vivo* analysis identifies effective HIV-1 latency–reversing drug combinations. J. Clin. Invest. 125, 1901–1912. 10.1172/JCI80142 25822022 PMC4463209

[B26] Laspia M. F. Rice A. P. Mathews M. B. (1989). HIV-1 Tat protein increases transcriptional initiation and stabilizes elongation. Cell 59, 283–292. 10.1016/0092-8674(89)90290-0 2553266

[B27] Lopez A. P. Kugelman J. R. Garcia-Rivera J. Urias E. Salinas S. A. Fernandez-Zapico M. E.. (2016). The structure-specific recognition protein 1 associates with lens epithelium-derived growth factor proteins and modulates HIV-1 replication. J. Mol. Biol. 428, 2814–2831. 10.1016/j.jmb.2016.05.013 27216501 PMC4938748

[B28] Mousseau G. Clementz M. A. Bakeman W. N. Nagarsheth N. Cameron M. Shi J.. (2012). An analog of the natural steroidal alkaloid cortistatin A potently suppresses Tat-dependent HIV transcription. Cell Host Microbe 12, 97–108. 10.1016/j.chom.2012.05.016 22817991 PMC3403716

[B29] Mousseau G. Kessing C. F. Fromentin R. Trautmann L. Chomont N. Valente S. T. (2015). The Tat inhibitor didehydro-cortistatin A prevents HIV-1 reactivation from latency. MBio 6:e00465–e00415. 10.1128/mBio.00465-15 26152583 PMC4495168

[B30] Nabel G. Baltimore D. (1987). An inducible transcription factor activates expression of human immunodeficiency virus in T cells. Nature 326, 711–713. 10.1038/326711a0 3031512

[B31] O'Connor C. M. Nukui M. Gurova K. V. Murphy E. A. (2016). Inhibition of the FACT complex reduces transcription from the human cytomegalovirus major immediate early promoter in models of lytic and latent replication. J. Virol. 90, 4249–4253. 10.1128/JVI.02501-15 26865717 PMC4810550

[B32] Ott M. Geyer M. Zhou Q. (2011). The control of HIV transcription, keeping RNA polymerase II on track. Cell Host Microbe 10, 426–435. 10.1016/j.chom.2011.11.002 22100159 PMC3478145

[B33] Pace M. J. Agosto L. Graf E. H. O'Doherty U. (2011). HIV reservoirs and latency models. Virology 411, 344–354. 10.1016/j.virol.2010.12.041 21284992 PMC3618966

[B34] Power D. Santoso N. Dieringer M. Yu J. Huang H. Simpson S.. (2015). IFI44 suppresses HIV-1 LTR promoter activity and facilitates its latency. Virology 481, 142–150. 10.1016/j.virol.2015.02.046 25776761 PMC4437885

[B35] Ruelas D. S. Greene W. C. (2013). An integrated overview of HIV-1 latency. Cell 155, 519–529. 10.1016/j.cell.2013.09.044 24243012 PMC4361081

[B36] Shan L. Deng K. Shroff N. S. Durand C. M. Rabi S. A. Yang H.-C.. (2012). Stimulation of HIV-1-specific cytolytic T lymphocytes facilitates elimination of latent viral reservoir after virus reactivation. Immunity 36, 491–501. 10.1016/j.immuni.2012.01.014 22406268 PMC3501645

[B37] Walker-Sperling V. E. Pohlmeyer C. W. Tarwater P. M. Blankson J. N. (2016). The effect of latency reversal agents on primary CD8+ T cells: implications for shock and kill strategies for human immunodeficiency virus eradication. EBioMedicine 8, 217–229. 10.1016/j.ebiom.2016.04.019 27428432 PMC4919475

[B38] Wong J. K. Hezareh M. Günthard H. F. Havlir D. V. Ignacio C. C. Spina C. A.. (1997). Recovery of replication-competent HIV despite prolonged suppression of plasma viremia. Science 278, 1291–1295. 10.1126/science.278.5341.1291 9360926

[B39] Yukl S. A. Shergill A. McQuaid K. Gianella S. Lampiris H. Hare C. B.. (2010). Effect of raltegravir-containing intensification on HIV burden and T cell activation in multiple Gut sites of HIV+ adults on suppressive antiretroviral therapy. AIDS 24:2451. 10.1097/QAD.0b013e32833ef7bb 20827162 PMC2997807

[B40] Zhu J. Gaiha G. D. John S. P. Pertel T. Chin C. R. Gao G.. (2012). Reactivation of latent HIV-1 by inhibition of BRD4. Cell Rep. 2, 807–816. 10.1016/j.celrep.2012.09.008 23041316 PMC3523124

