# Supplementary figures and images for: Curaxin CBL0100 Blocks HIV-1 Replication and Reactivation through Inhibition of Viral Transcriptional Elongation
Source: Front Microbiol. 2017 Oct 17;8:2007. doi: 10.3389/fmicb.2017.02007 (PMC5651003; doi:10.3389/fmicb.2017.02007)

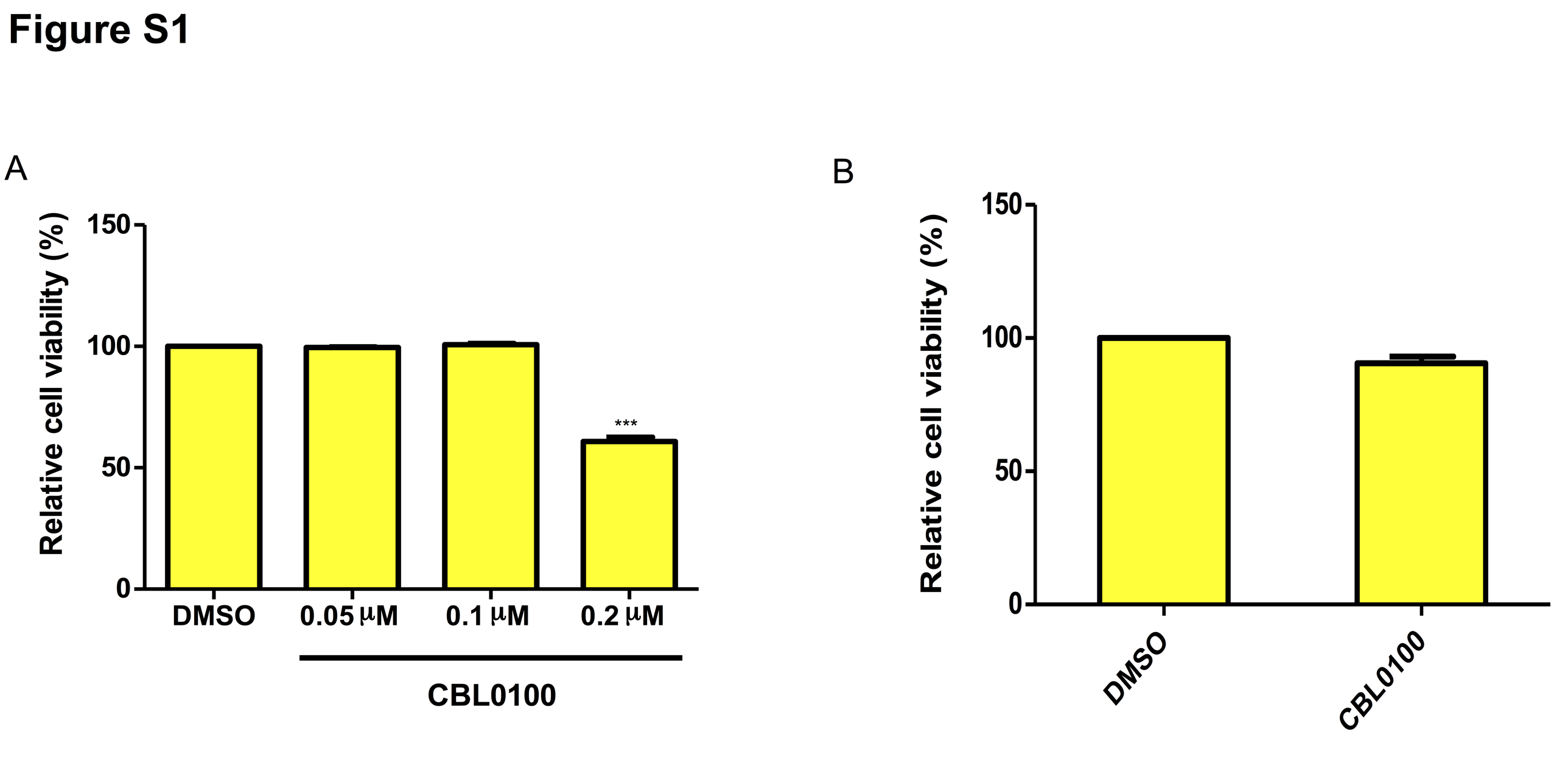

Supplement: Figure S1 — (A) The cytotoxicity test of CBL0100 at three selected concentrations (0.05, 0.1, and 0.2 μM). Data was obtained from two independent experiments and compared to DMSO (mean ± s.e.m., *p < 0.05, **p < 0.01, ***p < 0.001, student t-test). (B) Cell viability of healthy primary CD4+ T cells treated with CBL0100 at 0.1 μM for 3 days. Data is normalized to DMSO control (mean ± s.d., *p < 0.05, **p < 0.01, ***p < 0.001, student t-test). [file Image1.TIFF]

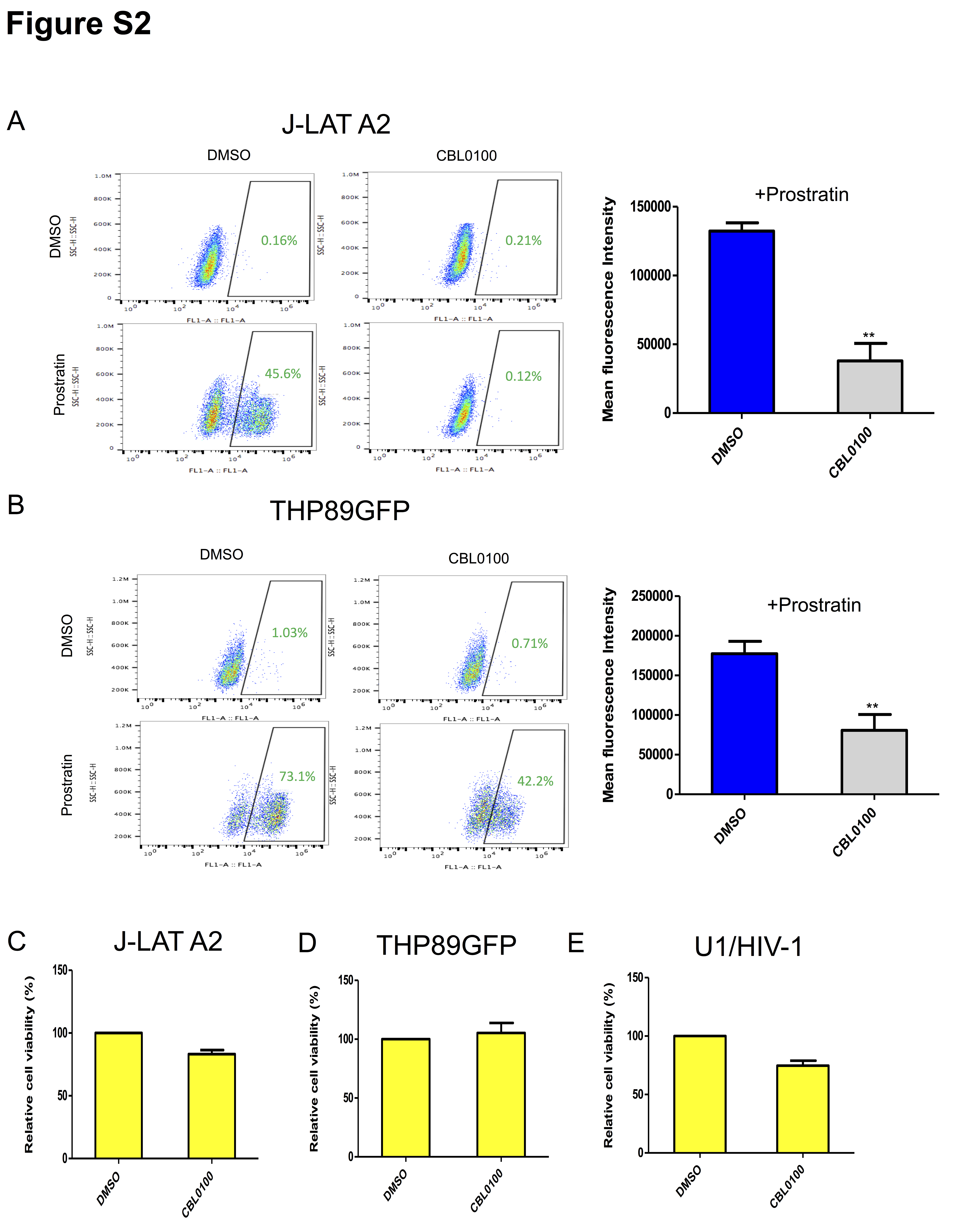

Supplement: Figure S2 — (A) J-LAT-A2 cells were stimulated with prostratin (1 μM) or mock treated with DMSO in the presence of CBL0100 (0.1 μM) or DMSO for 24 h. The percentage of GFP+ cells was measured by flow cytometry. MFI of GFP+ cells was also measured. Data are from three independent experiments (mean ± s.e.m., *p < 0.05, **p < 0.01, ***p < 0.001, Student t-test). (B) The same experiment as in panel A was performed for THP89GFP cells. 0. 5 μM Prostratin and 0.2 μM CBL0100 were used. (C) Cell viability of J-LAT A2 following 24-h treatment with 0.1 μM CBL0100. Data is normalized to DMSO control and from three independent experiments (mean ± s.e.m., *p < 0.05, **p < 0.01, ***p < 0.001, student t-test). (D) The same experiment as in panel C was performed for THP89GFP cells. 0.2 μM CBL0100 were used. (E) The same experiment as in (C) was performed for U1/HIV-1 cells. [file Image2.TIFF]

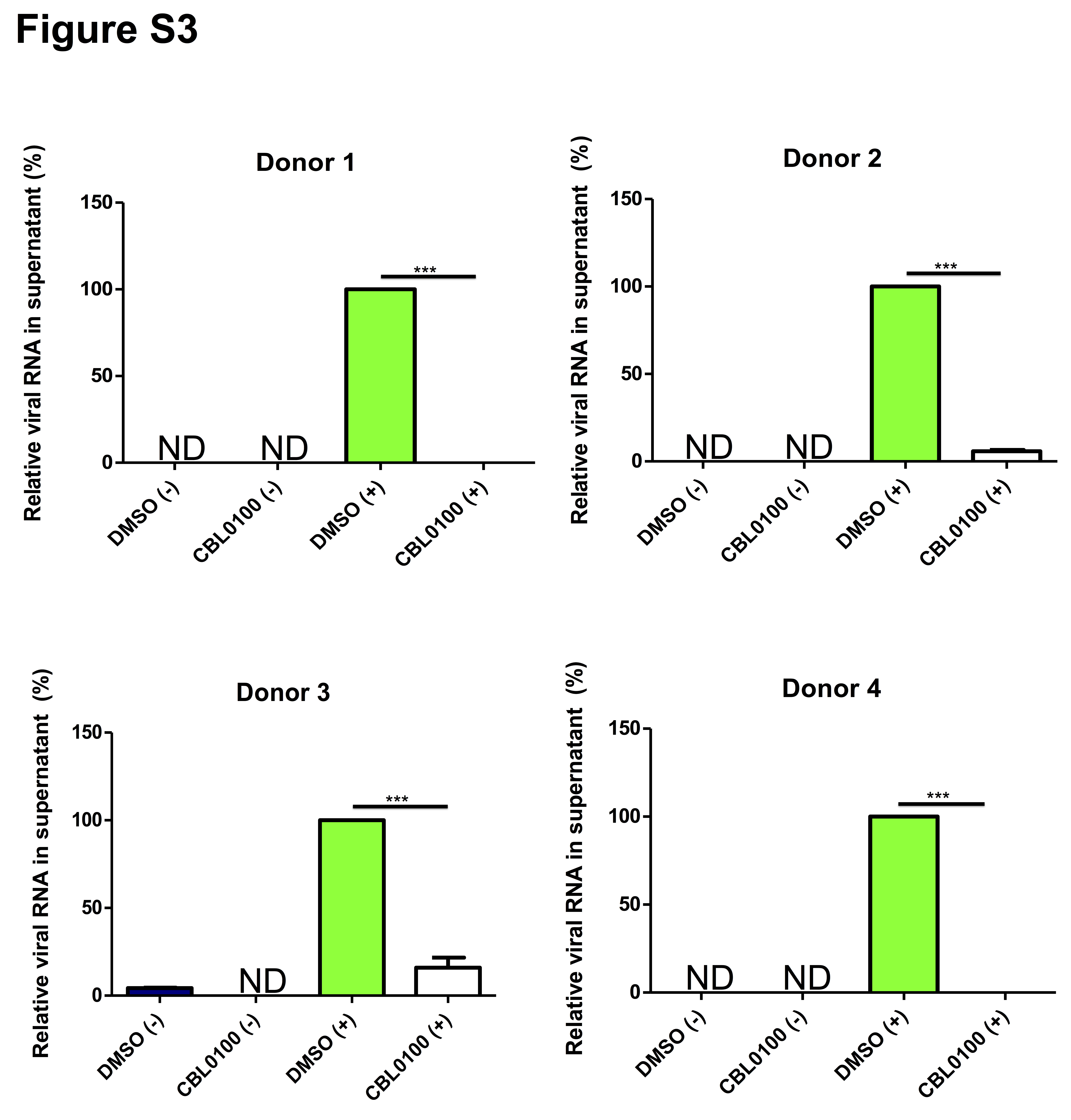

Supplement: Figure S3 — CD8-depeleted PBMCs were stimulated with anti-CD3/CD28 (+) or mock treated with PBS (–) in the presence of 0.1 μM CBL0100 or DMSO for 3 days. The viral RNA level in the supernatant from all tested samples was determined by qPCR and represented as relative to DMSO (+) for respective donors. Effect of CBL0100 on individual donors was measured from triplicate measurements (mean ± s.d., *p < 0.05, **p < 0.01, ***p < 0.001, student t-test). ND, Not detected. [file Image3.TIFF]

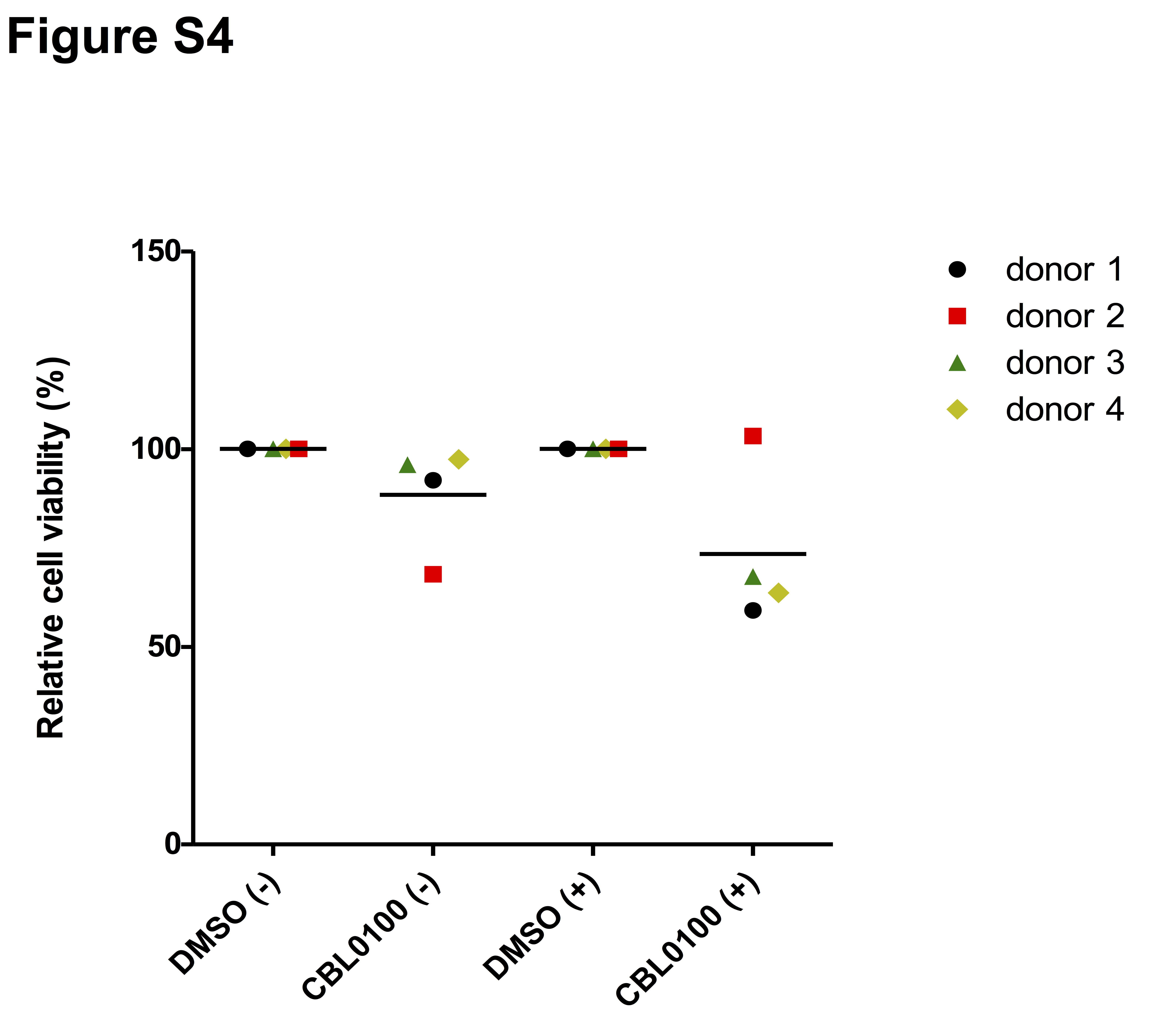

Supplement: Figure S4 — Cell viability of CD8-depeleted PBMCs from four HIV-positive, cART-treated donors following the CBL0100 treatment with the anti-CD3/CD28 antibodies induction (+) or the mock treatment (–). ATP levels was assessed for each conditions and data was normalized to the corresponding DMSO control. [file Image4.TIFF]
