# Supplementary material for: Curaxin CBL0100 Blocks HIV-1 Replication and Reactivation through Inhibition of Viral Transcriptional Elongation
Source: Front Microbiol. 2017 Oct 17;8:2007. doi: 10.3389/fmicb.2017.02007 (PMC5651003; doi:10.3389/fmicb.2017.02007)
Supplement: Table S1 — Primer sequences for the qPCR assays. [file Table1.docx]

| Primers | Sequences |
| --- | --- |
| LTR/Nuc1-for^1^ | 5’-CTG GGA GCT CTC TGG CTA ACT A-3’ |
| LTR/Nuc1-rev^1^ | 5’-TTA CCA GAG TCA CAC AAC AGA CG-3’ |
| Initiation-for^2^ | 5’- GTT AGA CCA GAT CTG AGC CT-3’ |
| Initiation-rev^2^ | 5’-GTG GGT TCC CTA GTT AGC CA-3’ |
| Elongation 1-for^2^ | 5’-TGG GAG CTC TCT GGC TAACT-3’ |
| Elongation 1-rev^2^ | 5’-TGC TAG AGA TTT TCC ACA CTG A-3’ |
| Elongation 2-for^2^ | 5’-GTA ATA CCC ATG TTT TCA GCA TTA TC-3’ |
| Elongation 2-rev^2^ | 5’-TCT GGC CTG GTG CAA TAG G-3’ |
| P1^1^ | 5’-ATGCCACGTAAGCGAAACTCTGGGTCTCTCTGGTTAGAC-3’ |
| P2^1^ | 5’-CCATCTCTCTCCTTCTAGC-3’ |
| P3^1^ | 5’-ATGCCACGTAAGCGAAACT-3’ |
| P4^1^ | 5’-CTGAGGGATCTCTAGTTACC-3’ |
| Probe^1^ | 5’-56-FAM/CACTCAAGGCAAGCTTTATTGAGGC/36-TAMSp-3’ |
| GAPDH-for^2^ | 5'- GCC TCT TGT CTC TTA GAT TTG GTC -3’ |
| GAPDH-rev^2^ | 5'- TAG CAC TCA CCA TGT AGT TGA GGT -3’ |
| Gag_for^2^ | 5’-GAC GCT CTC GCA CCC ATC TC-3’ |
| Gag_rev^2^ | 5’-CTG AAG CGC GCA CGG CAA-3’ |

**Table S1**

1. **Mousseau G, Kessing CF, Fromentin R, Trautmann L, Chomont N, Valente ST.** 2015. The Tat inhibitor didehydro-cortistatin A prevents HIV-1 reactivation from latency. MBio **6:**e00465-00415.
2. **Zhu J, Gaiha GD, John SP, Pertel T, Chin CR, Gao G, Qu H, Walker BD, Elledge SJ, Brass AL.** 2012. Reactivation of latent HIV-1 by inhibition of BRD4. Cell reports **2:**807-816.
